# Golden Flower Tibetan Tea Polysaccharides Alleviate Constipation in Mice by Regulating Aquaporins-Mediated Water Transport System and Gut Microbiota

**DOI:** 10.3390/foods13172749

**Published:** 2024-08-29

**Authors:** Manyou Yu, Jiayuan Zhao, Qingling Xie, Junlin Deng, Yongqing Zhu, Jian Chen, Zhuoya Xiang, Ting Zhang, Gang Liu, Chen Xia, Liugang Shi, Bin Wu, Irene Gouvinhas, Ana Novo Barros

**Affiliations:** 1 Institute of Agro-Products Processing Science and Technology (Institute of Food Nutrition and Health), Sichuan Academy of Agricultural Sciences, Chengdu 610066, China; jiangymy518@163.com (M.Y.); xxxieqingling@163.com (Q.X.); junlinr@scsaas.cn (J.D.); xiachen3722496@163.com (Y.Z.); nkyjgsgnsp@163.com (J.C.); xiangzhuoya2015@163.com (Z.X.); 2 Centre for the Research and Technology of Agro-Environmental and Biological Sciences (CITAB)/Institute for Innovation, Capacity Building and Sustainability of Agri-Food Production (Inov4Agro), University of Trás-os-Montes and Alto Douro (UTAD), 5000-801 Vila Real, Portugal; igouvinhas@utad.pt (I.G.); abarros@utad.pt (A.N.B.); 3 College of Life Science, Sichuan Normal University, Chengdu 610101, China; jiangnanyu123@126.com; 4 Tea Research Institute, Sichuan Academy of Agricultural Sciences, Chengdu 610066, China; zhangting8608@163.com; 5 Yazhou Hengtai Tea Industry Co., Ltd., Ya’an 625100, China; liugangshi1981@outlook.com (L.S.); wubin660701@gmail.com (B.W.)

**Keywords:** tea polysaccharides, mice constipation, gut microbiota

## Abstract

Constipation, a widespread gastrointestinal disorder, often leads to the exploration of natural remedies. This study examines the efficacy of Golden Flower Tibetan Tea Polysaccharides (GFTTPs) in alleviating constipation in mice. Chemical analyses reveal that GFTTPs possess O-H, carboxyl, carboxylic acid (-COOH), and C-O-C groups, alongside a porous crystal structure with thermal stability. In animal experiments, GFTTPs significantly upregulated aquaporin 3 (AQP3) and aquaporin 8 (AQP8) expressions in the colon, enhancing water absorption and reducing fecal water content. At a 400 mg/kg dosage, GFTTPs notably improved colonic tissue alterations and serum levels of excitatory neurotransmitters caused by loperamide hydrochloride. They also beneficially altered gut microbiota, increasing *Coprococcus*, *Lactobacillus*, and *Pediococcus* populations. These changes correlated with improved stool frequency, consistency, and weight in constipated mice. Importantly, GFTTPs at 200 and 400 mg/kg doses exhibited comparable effects to the normal control group in key parameters, such as gastrointestinal transit rate and fecal moisture. These findings suggest that GFTTPs may serve as a potent natural remedy for constipation, offering significant therapeutic potential within the context of gut health and with promising implications for human applications.

## 1. Introduction

Tea, a beverage treasured for centuries, continues to hold around the world [1]. Commercially, it is available in five major varieties, distinguished by their unique fermentation processes [2]. Among these, dark tea is a post-fermented type that has been appreciated since the era of China’s Ming Dynasty, around 1500 A.D. [3,4]. This tea undergoes microbial-mediated pile fermentation, resulting in diverse variants, among which Ya’an Tibetan tea is particularly noteworthy [2].

Extensive scientific research has unveiled its broad spectrum of health benefits, including antioxidative [5], cytoprotective [6], radiation-damage-mitigating [7], blood-pressure-regulating [2], lipid-lowering [8], and atherosclerosis-reducing properties [9]. Moreover, Ya’an Tibetan tea exhibits notable anti-inflammatory effects, primarily by modulating gut microbiota composition and regulating inflammation and immune pathways [10].

Golden Flower Tibetan Tea (GFTT) is an innovative variant derived from the traditional process of Ya’an Tibetan tea [11]. The formation of GFTT undergoes a series of steps with fresh tea leaves, including killing, kneading, smothering, drying, selecting, stacking, blending, tightening, and flowering. The end product is dominated by the microbe *Eurotium cristatum*, which provides the characteristic “flowering” effect following deep fermentation, thereby enhancing both the flavor and health-promoting attributes of the traditional Tibetan tea [12].

Originating from the rugged terrain of southwestern China, Ya’an Tibetan tea has carved a unique place in the history and culture of Tibet, navigating through its challenging mountainous landscapes [2,4]. Over time, it has emerged as an indispensable elixir, deeply ingrained in the daily lives of millions of Tibetan individuals. Despite the known benefits of Tibetan tea, the specific mechanisms through which its polysaccharides alleviate constipation, particularly in relation to water transport and gut microbiota modulation, remain underexplored. An intriguing observation arises from the dietary habits of Tibetan nomads, who endure prolonged periods, spanning up to eight months or even the entire year, without consuming vegetables or fruits [13]. Remarkably, despite this dietary restriction, these individuals display no overt signs of constipation. A recent study by Bai et al. (2022) [14] highlighted that the insoluble dietary fiber derived from tea processing exhibited notable ameliorative effects on slow transit constipation [15]. Furthermore, the underlying mechanism through which Fu brick tea aqueous extract alleviated constipation was found to involve the regulation of aquaporins, which are key mediators of water transport, in conjunction with the gut microbiota [3,14]. Notably, tea polysaccharides were identified as potentially functional components contributing to these effects [16], which are a combination of polysaccharides and proteins. And the fraction of polysaccharides are either linear or branched polymers of monosaccharides linked by glycosidic bonds [17].

However, the current literature lacks a comprehensive understanding of the physicochemical properties and specific mechanisms of action of these polysaccharides, particularly in the context of constipation relief. The study of Golden Flower Tibetan Tea Polysaccharides (GFTTPs) presents significant relevance to the field of food science, particularly in the development of functional foods that promote gastrointestinal health. Functional foods, which are designed to provide health benefits beyond basic nutrition, have gained considerable attention in recent years for their role in preventing and managing chronic conditions, such as gastrointestinal disorders. This study aims to address these research gaps by investigating the physicochemical properties and structural characteristics of GFTTP. Additionally, we explore the expression of genes related to the water transport system, gut microbiota, and short-chain fatty acid metabolism in mice, with the goal of providing insights that could guide the dietary management of constipation using GFTTP. By investigating the mechanisms on the regulation of aquaporin-mediated water transport and the modulation of gut microbiota, our study may contribute to the broader understanding of how natural compounds can be utilized to develop innovative dietary interventions aimed at improving gut function and alleviating conditions like constipation.

## 2. Materials and Methods

The experimental design of this study was carefully constructed to investigate the therapeutic effects of Golden Flower Tibetan Tea Polysaccharides (GFTTPs) on constipation, with a particular focus on their role in regulating aquaporin-mediated water transport and gut microbiota. These mechanisms are directly related to gastrointestinal health, which is a key area of interest in the field of functional foods and nutritional science.

### 2.1. Materials and Chemicals

The Golden Flower Tibetan Tea (GFTT) was provided by Yazhou Hengtai Tea Company in Mingshan District, Ya’an City, Sichuan Province. Enzymes including α-amylase, glucoamylase, and alcalase were obtained from Solarbio Science & Technology Co., Ltd. (Beijing, China). Chemicals: rutin, ethanol, dimethyl sulfoxide (DMSO), ascorbic acid, potassium persulfate, sodium dihydrogen phosphate, sodium hydrogen phosphate, sodium chloride, potassium ferrocyanide, ferric chloride, 2,2′-azino-bis(3-ethylbenzothiazoline-6-sulfonic acid) (ABTS), trichloroacetic acid (TCA), and 2,2-diphenyl-1-picrylhydrazyl (DPPH), all extra pure (>99%) were purchased from Meryer Biochemical Technology Co., Ltd. (Shanghai, China). Loperamide hydrochloride (LOP) and picric acid (PA) were purchased from Cologne Chemicals Co., Ltd. (Chengdu, China). Tablets of phenolphthalein (PHE, 0.1 g per tablet) were provided by Shandong Renhetang Pharmaceutical Co., Ltd. (Linyi, China). Standards involving gallic acid (GA), glucose, bovine serum albumin (BSA), and short-chain fatty acids (SCFAs) were obtained from Sigma (St. Louis, MO, USA). All other chemicals and reagents used in the experiment were of analytical grade.

### 2.2. Preparation of Golden Flower Tibetan Tea Polysaccharides (GFTTP)

To prepare GFTTP, 1 kg of GFTT was subjected to three rounds of hot water extraction (75 °C, 1:10 material-to-liquid ratio) for 2 h, 1 h, and 30 min, respectively. The resulting extract was concentrated at 50 °C to one-fifth of its original volume, then combined with anhydrous ethanol (1:4 ratio) for 24 h precipitation.

The collected precipitate was then dissolved in 1500 mL of distilled water and digested with 0.5 g of α-amylase at 90 °C for 2 h. After cooling to 60 °C, the pH was adjusted to 10.5 using 1 M NaOH. It was further digested with 0.5 mL of alkaline protease at 60 °C for 1.5 h. The pH was then adjusted to around 4.25 using HCl, and 0.5 g of glycosylase was added for 4 h digestion.

The mixture was boiled for 5 min and then precipitated with a four-fold volume of ethanol. After a 12 h resting period at 4 °C, the precipitate was collected, washed thrice with anhydrous ethanol, and freeze-dried, yielding 76.84 g of GFTTP. The obtained GFTTPs were kept at −4 °C for further use.

### 2.3. Chemical Characterization of GFTTPs

#### 2.3.1. Determination of Molecular Weight

The molecular weight (Mw) of GFTTPs was evaluated using a High-Performance Liquid Chromatography (HPLC, Agilent 1100-AB 400, Shimadzu Corp., Tokyo, Japan) system equipped with gel permeation (GPC, a TSKgel G4000 PWXL column, 7.8 mm × 300 mm, Tosoh Crop., Tokyo, Japan) and an evaporative light scattering detector (ELSD), using phosphate buffer (pH 7.0) as mobile phase. The flow rate was 1.0 mL/min, and the column temperature was set at 30 °C [18].

#### 2.3.2. Measurement of Total Sugar, Phenols, and Proteins

The total sugar content of GFTTPs was measured using the 3,5-dinitrosalicylic acid (DNS) colorimetric method [19] and expressed in milligrams of glucose per gram (mg glucose/g). The total phenol content was determined using the Folin–Ciocalteu colorimetric method [16] and expressed as milligrams of GA per gram (mg GA/g). The total protein content was evaluated using the Coomassie Brilliant Blue method [18] and expressed as milligrams of BSA per gram (mg BSA/g).

#### 2.3.3. Evaluation of Monosaccharide Composition

The monosaccharide composition of GFTTPs was analyzed using 1-phenyl-3-methyl-5-pyrazolone (PMP) pre-column derivatization–HPLC method [17]. In brief, 15 mg of GFTTPs were hydrolyzed with 5 mL of trifluoroacetic acid (TFA, 2.0 M) in the 20 mL Crimp Neck Vial at 100 °C for 2 h. Then, the trifluoroacetic acid was removed by repeated co-distillations with methanol under reduced pressure. The hydrolysate was dissolved in 1 mL of NaOH solution (0.3 M), and then derivatized by PMP (0.5 M) at 70 °C for 2 h. After being neutralized by HCl (0.3 M) and extracted twice by chloroform, the effluent phase was subsequently filtered by microporous membrane for HPLC analysis on an Agilent 1100 instrument (Shimadzu Corp., Tokyo, Japan), equipped with Eclipse Plus C18 column (4.6 mm × 250 mm, 5 μm, Shimadzu Corp., Tokyo, Japan). The column temperature was set to 30 °C. The mobile phase consisted of PBS (100 mM, pH 6.7) (Solvent A) and acetonitrile (Solvent B). The linear gradient program (t in min—%A) was as follows: t = 0–86%; t = 9–83%; t = 28–78%; t = 29–50%; t = 31–50%; t = 32–86%; t = 36–86%. The flow rate was 1.0 mL/min, and the detection wavelength was 250 nm. The monosaccharides of GFTTPs were identified by comparing the retention times of the peaks with those of the standards.

#### 2.3.4. Fourier-Transform Infrared (FT-IR) Spectroscopy

The FT-IR spectra of GFTTPs were obtained via KBr pellet pressing [20]. In short, 2 mg of polysaccharides and approximately 200 mg of KBr were homogenized and pressed, and then scanned at a range of 1000–4000 cm^−1^ using a Thermo Scientific Nicolet iS-5 spectrometer (Thermo Fisher Scientific Inc., Waltham, MA, USA). FT-IR spectra were recorded on a Nicolet IS-10 spectrometer (Thermo Fisher Scientific Inc., Waltham, MA, USA) in the 1000–4000 cm^−1^ wavenumber region, using a 16-scan-per-sample cycle and a resolution of 4 cm^−1^.

#### 2.3.5. X-ray Diffraction (XRD) Analysis

X-ray diffraction (XRD) measurement was carried out on a RIGAKU Ultima IV X-ray diffractometer (Rigaku Corporation, Tokyo, Japan) with Cu Kα filtered radiation (λ = 1.54 Å) in the scanning range of 5–90°. The accelerating voltage and current used were 40 kV and 40 mA, respectively.

#### 2.3.6. Thermogravimetric Analysis (TGA)

TGA was monitored using a Netzsch TG 209 F1 thermogravimetric analyzer (NETZSCH-Gerätebau GmbH, Selb, Germany) from 25 to 800 °C with a heating rate of 10 °C/min under a nitrogen atmosphere (25 mL/min).

#### 2.3.7. Scanning Electron Microscopy (SEM)

The morphologies of the GFTTP hydrogels were observed using a Zeiss Sigma 300 microscope (Carl Zeiss AG, Oberkochen, Germany) at an acceleration voltage of 10 kV. Prior to SEM imaging, the freeze-dried samples were cut into small pieces, mounted on a metal stub with a conductive tape and sputter-coated with a thin gold layer. The average pore size of the hydrogels was measured using the Nano Measurer 1.2.5 (Fudan University, Shanghai, China).

### 2.4. Evaluation of Relieving Effect of GFTTPs in Constipated Mice

#### 2.4.1. Animal Experiments

The animal experiments were performed according to the guidelines of the Institutional Animal Ethics Committee and were approved by the Institutional Animal Committee of Sichuan Normal University (Approval code: 2024LS055, Approval date: 5 January 2024). A total of 48 male Kunming mice (specific-pathogen-free grade, aged 6 weeks, weighing 20 ± 2 g) were purchased from Chengdu Dashuo Experimental Animal Co., Ltd. (Chengdu, China). All animals were kept under standard conditions with a light/dark cycle of 12/12 h, temperature of 25 ± 2 °C, and relative humidity of 60 ± 5%. They had free access to water and were fed standard commercial mouse food.

**Figure 1:**
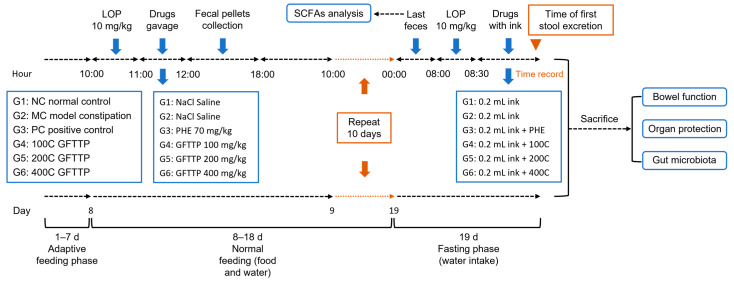
Diagram of animal experimental design.

The experimental design is shown in Figure 1. After adaptive feeding for 1 week, the mice were randomly divided into six groups (*n* = 8 per group) in separate cages. Five groups were induced with 10 mg/kg i.g (intragastric administration) of LOP for constipated mice, and then after 1 h, each group was administrated with different treatments: Group 1, normal control with sterile saline (0.9% NaCl, 0.1 mL/10 g i.g) (G1: NC); Group 2, LOP-induced constipation model treated with saline (G2: MC); Group 3, LOP-induced positive control treated with PHE (70 mg/kg i.g) (G3: PC); Group 4, LOP-induced constipated mice treated with low-dose GFTTPs (100 mg/kg i.g) (G4: 100C); Group 5, LOP-induced constipated mice treated with middle-dose GFTTPs (200 mg/kg i.g) (G5: 200C); Group 6, LOP-induced constipated mice treated with high-dose GFTTPs (400 mg/kg i.g) (G6: 400C). After gavage with corresponding drugs for 1 h, all mice were moved into individual cages and were allowed to eat and drink at their will for 6 h. During this period, fecal pellets of mice were collected, numbered, and weighed (recorded as fecal wet weight W_0_), and then the fecal pellets were dried in a constant temperature drying oven at 105 °C to a constant weight (recorded as fecal dry weight W_1_). The fecal moisture was calculated as (W_0_ − W_1_)/W_0_. The same experimental procedure was repeated for 11 days.

On the 12th day, the separate animals were fasted overnight after the last food consumption (water intake allowed). During the fasting period, feces in the last instance were collected and stored in a refrigerator at −80 °C for subsequent analysis of intestinal microorganisms. After the fasting, mice from G2 to G6 were induced with 10 mg/kg of LOP by gavage. Thirty minutes later, the NC and CM groups were administrated with 0.2 mL of ink. Mice from the PC group were treated with 0.2 mL of ink mixed with PHE. The other three groups were fed with 0.2 mL of ink mixed with different dosages of GFTTPs (100, 200, 400 mg/kg i.g, respectively). After gavage, the time of first black stool excretion of each animal was recorded. Within another 30 min, all mice were sacrificed by carbon-dioxide-induced anesthesia. Blood serum and organ samples were collected and stored. The mesentery was separated, and a section of the intestinal segment from the pylorus to the ileocecum was carefully straightened to measure its length. The distance from the pylorus to the front of the ink was recorded as the “ink advancement length”, while the overall length of the small intestine was recorded as the “total length of small intestine”. The small bowel advancement rate was calculated as Small intestine advancement rate (%) = (ink advancement length/whole length of small intestine) × 100%.

Throughout the entire experiment, daily recording of the mice’s body weight was conducted. Additionally, observations were made regarding signs such as fur condition, fecal characteristics, and mental state to assess the well-being of the mice and track the experiment’s progress.

#### 2.4.2. Assessment of Neurotransmitters

The levels of gastrin (GAS), motilin (MTL), somatostatin (SS), substance P (SP), and vasoactive intestinal peptide (VIP) in the serum were measured according to the enzyme-linked immunosorbent assay (ELISA) kit instructions (Xinyu Biotechnology Co., Ltd., Shanghai, China). This kit used the double-antibody sandwich method to determine the level of related indicators in mouse serum. The absorbance values were recorded at 450 nm using a microplate reader (K6600-B, Kaiao, Beijing, China)

#### 2.4.3. Real-Time Quantitative PCR (RT-qPCR) Analysis

Total RNA in colon tissue was extracted using Trizol reagent (BioTeke, Beijing, China). The isolated RNA (500 ng) was reversely transcribed into cDNA using Reverse Transcriptase kits (Beyotime Bio, Shanghai, China). In this experiment, LightCycler 480 II fluorescence quantifier (Roche, Indianapolis, IN, USA) was used for fluorescence quantitative analysis. The 2^−ΔΔCT^ method was used to calculate the relative gene expression. The primer sequences are shown in Table S1.

#### 2.4.4. Histopathological Analysis

Colon tissues were preserved by fixation in 4% paraformaldehyde and subsequently embedded in paraffin. From these prepared specimens, sections of 5 μm thickness were carefully sliced and subjected to staining with hematoxylin and eosin (H&E) for the purpose of pathological analysis. The histological distinctions in the colon were then observed under a light microscope (Olympus DP73, Tokyo, Japan).

#### 2.4.5. Gut Microbiota Analysis

According to product instructions, the metagenome DNA from mice’s fecal content was obtained using a TruSeq Nano DNA LT Library Prep Kit (Illumina Inc., San Diego, CA, USA). The dry ice was deployed for packaging the extracted DNA samples, after which it was dispatched to the MiSeqPE300 platform (Allwegene Technology, Beijing, China) for microbial composition analysis. The V3–V4 variable regions of the 16S bacterial ribosomal RNA gene sequences were selected for amplification. The gene sequencing and data analysis were conducted using a previously described method.

#### 2.4.6. Determination of Short-Chain Fatty Acids (SCFAs)

Fecal samples (100 mg) were dissolved in 50 μL of 15% phosphoric acid, then 100 μL 125 μg/mL internal standard isocaproic acid solution and 400 μL ether were added, and then mixed well for 1 min. The mixture was centrifuged at 12,000 rpm for 10 min at 4 °C, then the supernatant was filtrated through a 0.22 μm microporous membrane and detected using a DGU-20A5R HPLC system equipped with a C-18 reverse-phase column (Shimadzu Corp., Tokyo, Japan).

The concentration of SCFAs in the cecal contents and standard solutions of acetic acid, propionic acid, butyric acid, isobutyric acid, valeric acid, isovaleric acid, and caproic acid were analyzed by HPLC, and their standard curves were constructed.

### 2.5. Statistical Analysis

The experimental data are presented as the mean ± standard deviation (SD). A one-way analysis of variance (ANOVA), followed by Duncan’s test, was used to evaluate differences between groups using SPSS Version 20.0 (SPSS Software, Chicago, IL, USA). Additionally, the R language with the DESeq2 package (version 1.30.1) and the clusterProfiler package (version 3.18.0) were employed to determine differential expression (DESeq) and Kyoto Encyclopedia of Genes and Genomes (KEGG) term differences. *p*-values < 0.05 were considered statistically significant.

## 3. Results and Discussion

### 3.1. Physicochemical Properties of GFTTP

#### 3.1.1. Basic Structures of GFTTP

Crude tea polysaccharides (TPSs) characteristically contain inter-conjugated carbohydrates, proteins, and polyphenols [21]. Similar results were found in this study, showing that the content of total sugar, total phenols, and total proteins in GFTTPs was 17.83%, 9.24%, and 8.22%, respectively (Table 1). The composition of crude TPS varies with processing methods, including extraction and drying, as the concentrations of protein and polyphenol in GFTTPs are different in crude TPS as reported by [22]. The molecular weight of GFTTPs was also measured, and Mw, Mn, and Mp were 70,989 Da, 24,227 Da, and 66,395 Da, respectively (Table 1). Moreover, the biological activities of TPSs are closely associated with their monosaccharides’ compositions [23]. In alignment with this, we analyzed the monosaccharide compositions of GFTTPs (Table 1). Du et al. (2016) [21] reviewed the carbohydrate constituents of TPS, listing glucose (Glc), galactose (Gal), arabinose (Ara), rhamnose (Rha), xylose (Xyl), galacturonic acid (GalA), mannose (Man), ribose (Rib), and glucuronic acid (GulA). Our results show that the most abundant monosaccharides in GFTTPs were Ara, Gal, and GulA, which is consistent with the findings of Liu et al. (2023) [22].

#### 3.1.2. FT-IR Spectroscopy

**Figure 2:**
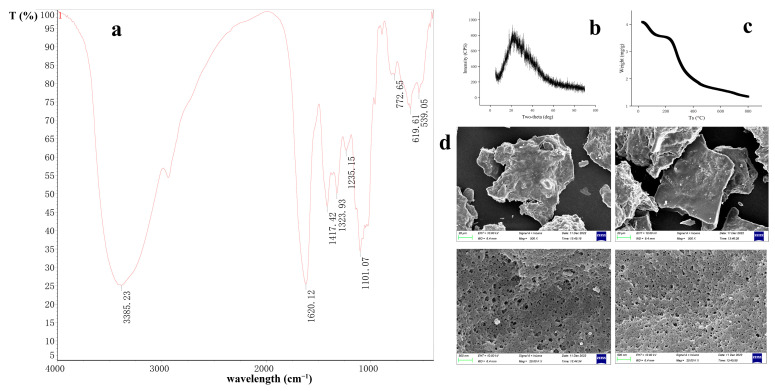
Chemical property of GFTTP. (**a**) FI-IR spectra of APP3a in the range of 400–4000 cm^−1^; (**b**) XRD analysis; (**c**) TGA assessment; (**d**) SEM.

Fourier-transform infrared (FT-IR) spectroscopy is a sophisticated analytical technique employed for the measurement of substance absorption of infrared light [24]. It finds extensive applications in characterizing the molecular structure, chemical bonding, and other pertinent information of various materials [14]. The FT-IR spectrum of GFTTP, as illustrated in Figure 2a, reveals distinct peaks at specific wavenumbers, offering valuable insights into the structural characteristics of GFTTPs. The prominent peak observed at 3385.23 cm^−1^ corresponds to the stretching vibration of the O-H group within the polysaccharides [24], confirming its presence in GFTTP. Furthermore, the peak detected at 1620.12 cm^−1^ indicates the presence of carboxyl and carboxylic acid groups (-COOH) [24], signifying that GFTTPs are acidic polysaccharides.

The peak at 1417.42 cm^−1^ can be attributed to the stretching vibration of subsidiary polysaccharides. The spectral region between 1417.42 cm^−1^ and 1235.15 cm^−1^ suggests the incorporation of glyoxylate within the polysaccharide structure. Another notable peak at 1101.07 cm^−1^ corresponds to the C-O-C stretching and variable angle vibration in the sugar ring, indicative of the presence of a pyranose ring. Finally, a minor peak at 772.65 cm^−1^ suggests the existence of an α-glycosidic bond within GFTTPs.

#### 3.1.3. XRD Analysis

X-ray diffraction (XRD) analysis is a powerful technique used to investigate the structural and crystalline properties of materials; the sharpness and intensity of peaks observed in XRD patterns provide insights into the crystallinity and internal structure of the analyzed material [25]. In general, well-defined and intense peaks in XRD patterns indicate a high degree of crystallinity, which often correlates with the mechanical traits, such as tensile strength and hardness, of the material. Conversely, materials with low crystallinity may exhibit altered physical and chemical properties, impacting factors like modified water retention, swelling behavior, and oil retention capacity of TPS.

The XRD pattern of GFTTPs, shown in Figure 2b, illustrates prominent peaks, indicating a high level of crystallinity in GFTTPs. Notably, significant and sharp signal peaks are observed within the range of 20° to 30°, with an absence of any significant extraneous peaks. GFTTPs possess a crystallinity of 11.74% and its molecular chains are arranged in an orderly manner, contributing to its well-defined crystal structure.

#### 3.1.4. TGA Analysis

Thermogravimetric analysis (TGA) is a widely used technique for investigating the thermal stability of substances [26]. It sheds light on the mass variations under processes such as dehydration, decomposition, and oxidation at different temperatures [27]. Figure 2c displays the thermal stability analysis of GFTTP, elucidating its response to varying temperatures. The TGA curve exhibits three distinct stages of heat loss. In the first stage (0–200 °C), a substantial weight change is observed, primarily attributed to the volatilization of free water. The second stage (200–250 °C) is characterized by a gradual decline in weight loss, indicating a diminished heat loss primarily associated with bound water. At higher temperatures, the polysaccharide undergoes decomposition, transforming into oligosaccharide substances and further degrading into monosaccharides, accompanied by caramelization reactions. Notably, a significant inflection point is observed at 250 °C, highlighting a decrease in the thermal stability of the sample during this phase. In the third stage (250–800 °C), complex compounds within the sample undergo pyrolysis. TGA analysis reveals that the thermal stability of GFTTPs exhibits a more pronounced change at 250 °C.

#### 3.1.5. SEM Analysis

Scanning electron microscopy (SEM) is a powerful tool for analyzing materials at the nanometer and micrometer scales, providing detailed insights into the external structure and morphological characteristics of GFTTPs. Figure 2d presents electron microscopy images of GFTTPs, enabling a closer examination of its physical features. At a magnification of 500×, the SEM images reveal that GFTTPs exhibit a layered lamellar structure with an overall loose and irregular arrangement. When observed at a higher magnification of 10,000×, GFTTPs display a honeycomb-like structure with regular, smooth, and compact cavities. These observations imply the weak interactions among glycosidic bonds, polysaccharide inter-chain, and intra-chain hydrogen bonds within GFTTPs. Additionally, the molecular weight and degree of polymerization of GFTTPs are reduced, leading to smaller particle sizes and a more loosely packed molecular structure. These valuable insights highlight how the physical characteristics of GFTTPs are influenced by its intermolecular bond strengths and polysaccharide molecular weight.

### 3.2. GFTTPs Relieve LOP-Induced Constipation in Mice

Our study findings indicate that GFTTPs exhibited no significant adverse effects at the doses tested (100, 200, 400 mg/kg), as evidenced by the lack of observed toxicological symptoms, changes in body weight, or alterations in behavior throughout the experiment. Additionally, histopathological examination of the major organs, including the liver, kidney, and colon, showed no signs of tissue damage or inflammation, further supporting the safety of GFTTPs at these dosages.

When compared to established laxatives such as phenolphthalein, GFTTPs demonstrated a more favorable safety profile. Phenolphthalein, while effective, has been associated with several side effects, including electrolyte imbalance and dependency with prolonged use. In contrast, GFTTPs did not exhibit such side effects, making it a potentially safer alternative for long-term management of constipation. These findings are crucial in assessing the feasibility of GFTTPs as a therapeutic agent, particularly for populations that require gentle and sustained relief from constipation.

#### 3.2.1. Effect of GFTTPs on Body Weight, Stool-Related Parameters, and GI Transit Rate

**Figure 3:**
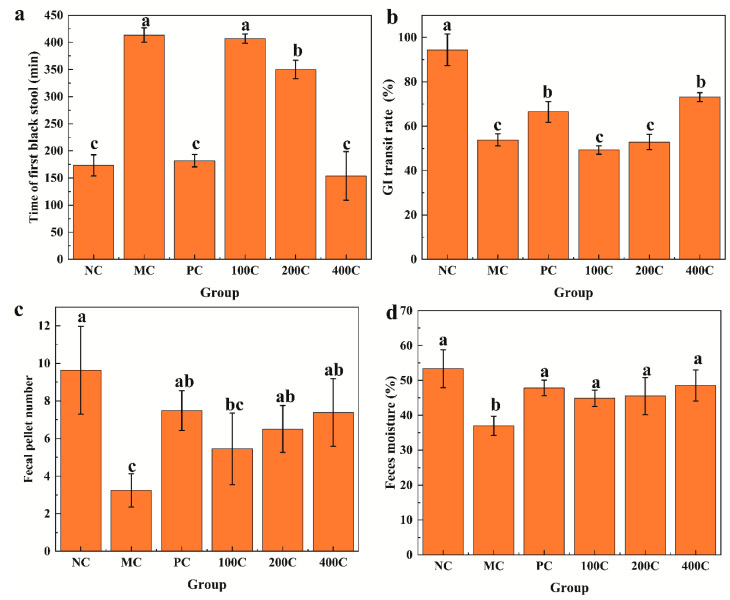
Effects of GFTTPs on physiological indexes in LOP-induced constipated mice. (**a**) Time of first black stool; (**b**) GI transit rate; (**c**) fecal pellet number; (**d**) feces moisture. Different letters indicate a significant difference according to Duncan’s multiple range test (*p* < 0.05) between all groups.

The effect of GFTTPs on the body weight gain of mice is shown in Figure S1. Notably, mice in the MC group displayed significantly lower weight gain than those in the NC, PC, 200C, and 400C groups (*p* < 0.05). This suggested that GFTTPs, at concentrations above 200 mg/kg, can relieve constipation-induced weight loss. In agreement, Zhang et al. (2022) [28] reported that constipation markedly reduced body weight compared with the control group. Generally, constipation leads to changes in various parameters, including the first dejection time, GI transit rate, fecal pellet number, and feces moisture [29]. After GFTTP administration, these indexes in mice were assessed (Figure 3). As expected, time of first black stool, GI transit rate, fecal pellet number, and feces moisture in NC did not significantly differ from the 200C or 400C treatments. Although the GI transit rates in both PC and 400C treatments were significantly lower than NC, they were significantly higher than that MC (*p* < 0.05). These results indicate that GFTTPs, particularly at 400 mg/kg, consistently showed significant improvements in stool frequency, consistency, and gastrointestinal transit time in constipated mice. These effects suggest that GFTTPs are effective in alleviating symptoms of constipation, likely through the modulation of aquaporin expression and gut microbiota. Complementing this, other studies found that functional fruit drinks and *Atractylodes macrocephala* polysaccharides improve these matrices significantly [30,31].

To further evaluate the therapeutic potential of GFTTPs, a comparative analysis of GFTTPs with established constipation treatments was conducted. GFTTPs were compared with phenolphthalein, a commonly used laxative, to evaluate its relative efficacy. While phenolphthalein acts primarily as a stimulant laxative, promoting bowel movements by irritating the intestinal lining, GFTTPs offer a multi-faceted mechanism of action, including the modulation of gut microbiota and the upregulation of aquaporins, which enhance water transport in the colon. Our results show that at the highest tested dose (400 mg/kg), GFTTPs were comparable to phenolphthalein in terms of improving gastrointestinal transit time and stool consistency. However, unlike phenolphthalein, GFTTPs did not cause significant alterations in serum electrolytes or induce dependency with prolonged use. Moreover, GFTTPs provided additional benefits by enhancing the populations of beneficial gut bacteria, such as *Lactobacillus* and *Pediococcus*, which are known to support overall gut health. These findings suggest that GFTTPs not only match the efficacy of conventional laxatives but also offer additional health benefits and a safer profile for long-term use.

#### 3.2.2. Effect of GFTTPs on Serum Levels of Gastrointestinal Regulatory-Related Peptides

**Figure 4:**
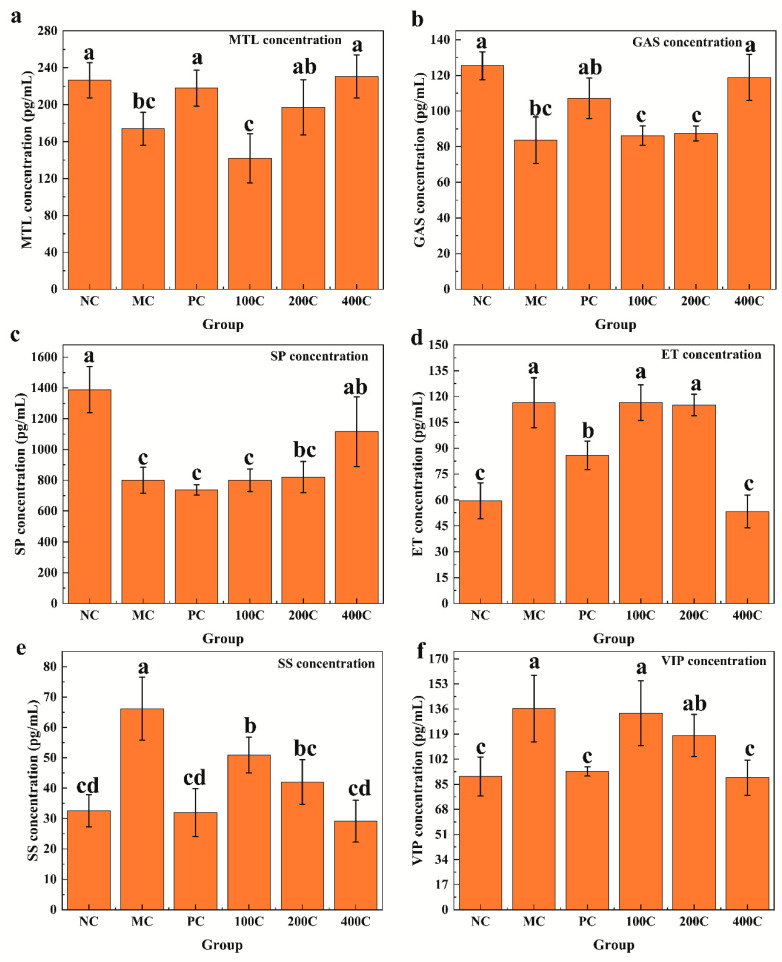
Effects of GFTTPs on levels of neurotransmitters. (**a**) gastrin (MTL); (**b**) gastrointestinal hormone (GAS); (**c**) substance P (SP); (**d**) endothelin (ET); (**e**) growth suppressor (SS); (**f**) vasoactive peptide (VIP). Different letters indicate a significant difference according to Duncan’s multiple range test (*p* < 0.05) between all groups.

Excitatory and inhibitory neurotransmitters play crucial roles in regulating defecation in mice by facilitating the contraction and relaxation of circular muscles, respectively [32]. In order to investigate the impact of GFTTPs on these constipation-linked neuropeptides, we analyzed the serum levels of gastrin (MTL), gastrointestinal hormone (GAS), substance P (SP), endothelin (ET), growth suppressor (SS), and vasoactive peptide (VIP) in constipated mice. The effects of GFTTPs on these neuropeptides are illustrated in Figure 4. Comparing with the MC group, we observed that the serum levels of excitatory neurotransmitters, namely MTL, GAS, and SP, significantly increased when treated with 400 mg/kg GFTTPs (*p* < 0.05), converging closely with the levels noted in the NC group. Previous studies demonstrated that gut hormones GAS and MTL play pivotal roles in regulating gastrointestinal motility. They are responsible for enhancing the secretion of gastric acid and pepsin, promoting the relaxation of pyloric sphincter, amplifying gastrointestinal motility, and expediting gastric emptying, collectively working towards the alleviation of constipation [29]. Additionally, as an endogenous neuropeptide, SP is known to stimulate the interstitial cells of Cajal (ICCs), inducing contractions in gastrointestinal smooth muscle and boosting gastrointestinal peristalsis [33]. These findings suggest that GFTTPs, at optimal doses, can alleviate loperamide hydrochloride-induced constipation by enhancing the synthesis of excitatory neurotransmitters.

However, turning focus to SS, it acts as an inhibitory neurotransmitter, inhibiting smooth muscle contraction and SP release, reducing gastrointestinal motility, and prolonging gastrointestinal transit time. Such actions generally intensify constipation symptoms [34]. Similarly, when the inhibitory neurotransmitter VIP is present in excess, it tends to relax the intestinal smooth muscle, slow intestinal motility, leading to constipation [35]. In contrast, the concentrations of ET, SS, and VIP in the 400C group were significantly higher than those in the MC group (*p* < 0.05), while no significant difference was observed compared to the NC group. These results indicate that GFTTPs modulate defecation in mice primarily by reducing the concentrations of inhibitory neurotransmitters.

#### 3.2.3. Effect of GTTTP on the Expression-Related Genes in the Colon

The gastrointestinal (GI) tract is a major organ involved in water transport, second only to the kidneys [36]. Water transport in the GI tract can occur through various means: passive diffusion, transcellular routes mediated by aquaporins (AQPs), co-transporters mechanisms, and strict paracellular pathways [37]. In the GI epithelium, AQPs, which are responsible for osmotically driven water transport, play a crucial role in both absorptive and secretive functions [38].

**Figure 5:**
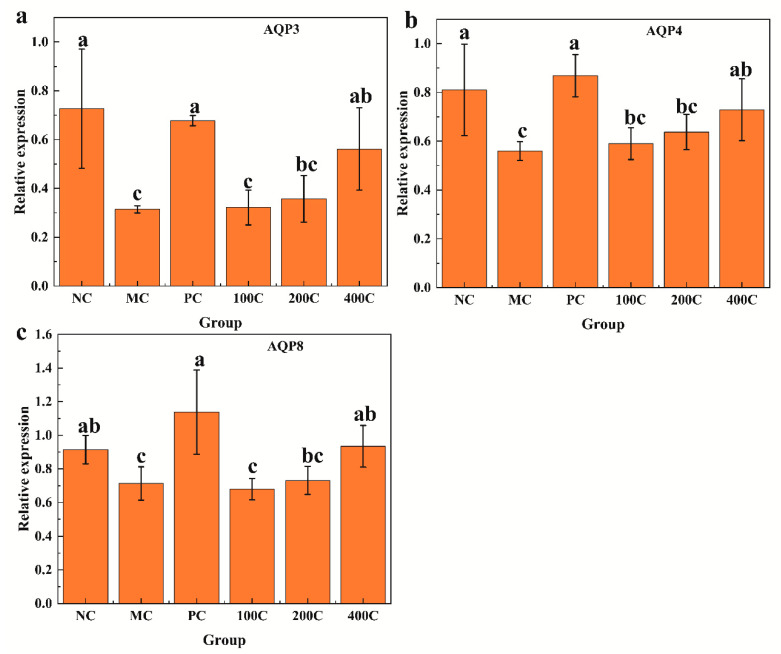
Effect of GTTTPs on the expression-related genes in the colon. (**a**) AQP3; (**b**) AQP4; (**c**) AQP8. Different letters indicate a significant difference according to Duncan’s multiple range test (*p* < 0.05) between all groups.

The expression levels of AQP3, AQP4, and AQP8 after administration with GFTTPs are shown in Figure 5a, 5b, and 5c, respectively. Comparing the MC group to the NC group, the expression of AQP3, AQP4, and AQP8 in colonic tissues was significantly reduced (*p* ≤ 0.05), indicating that ropivastigmine hydrochloride affects the expression of water channel proteins. The expression of these genes increased after administering 100 and 200 mg/kg GFTTPs, but these increments were not statistically significant when compared to the MC group. However, at a dosage of 400 mg/kg GFTTP, the expression of these genes significantly increased (*p* ≤ 0.05), approaching levels similar to the NC and PC groups. These findings suggest that GFTTPs can alleviate loperamide hydrochloride-induced constipation in mice by regulating the expression of genes encoding water channel proteins in colonic tissues. Our findings suggest that GFTTPs exert their effects on constipation not only through physical modulation of gut transit time and stool consistency, but also by influencing key biochemical pathways associated with water transport and gut microbiota. One of the primary mechanisms involves the upregulation of aquaporin proteins (AQP3 and AQP8) in the colon, which are critical for water transport across cell membranes. This upregulation facilitates increased water reabsorption, thereby reducing fecal water content and improving stool consistency.

GFTTPs exhibited a clear dose-dependent response in alleviating constipation, with the 400 mg/kg dose consistently showing the most significant improvements in stool frequency, consistency, and gastrointestinal transit time. The lower doses (100 mg/kg and 200 mg/kg), while effective to a degree, did not achieve the same level of efficacy, suggesting that the therapeutic benefits of GFTTPs increase with dosage. Our analysis suggests that 400 mg/kg is likely close to the optimal therapeutic dose for GFTTPs in this context. At this dose, GFTTPs not only provided the most pronounced relief from constipation, but also maintained a favorable safety profile, as evidenced by the absence of adverse effects observed during the study. The enhanced efficacy at higher doses may be attributed to more substantial modulation of gut microbiota and greater upregulation of aquaporins, which play critical roles in water transport and fecal moisture regulation. Further studies would be beneficial to confirm the optimal dose and to explore the dose–response relationship in greater detail, particularly in the context of long-term use.

The therapeutic effects of GFTTPs on constipation are closely linked to its ability to modulate water transport in the colon through the regulation of aquaporin proteins, particularly AQP3 and AQP8. Aquaporins are integral membrane proteins that facilitate water transport across cell membranes, playing a crucial role in maintaining water homeostasis within the gastrointestinal tract. Our study demonstrated that GFTTPs upregulate the expression of AQP3 and AQP8 in the colonic tissue of constipated mice, which correlates with improved stool consistency and enhanced gastrointestinal transit. The upregulation of these aquaporins likely facilitates increased water reabsorption, thereby reducing fecal water content and alleviating symptoms of constipation. This mechanism highlights the potential of GFTTPs as natural therapeutic agents that target specific molecular pathways involved in gastrointestinal function, providing a novel approach to managing constipation through dietary intervention. While our findings on the upregulation of aquaporins by GFTTPs are promising, the use of an animal model limits the direct applicability to humans. Variations in gut physiology and microbiota between species may influence these results. Future clinical studies are needed to confirm these effects in humans and to evaluate the long-term safety and efficacy of GFTTPs.

#### 3.2.4. Effect of GFTTPs on Histopathological Observation of Colon

**Figure 6:**
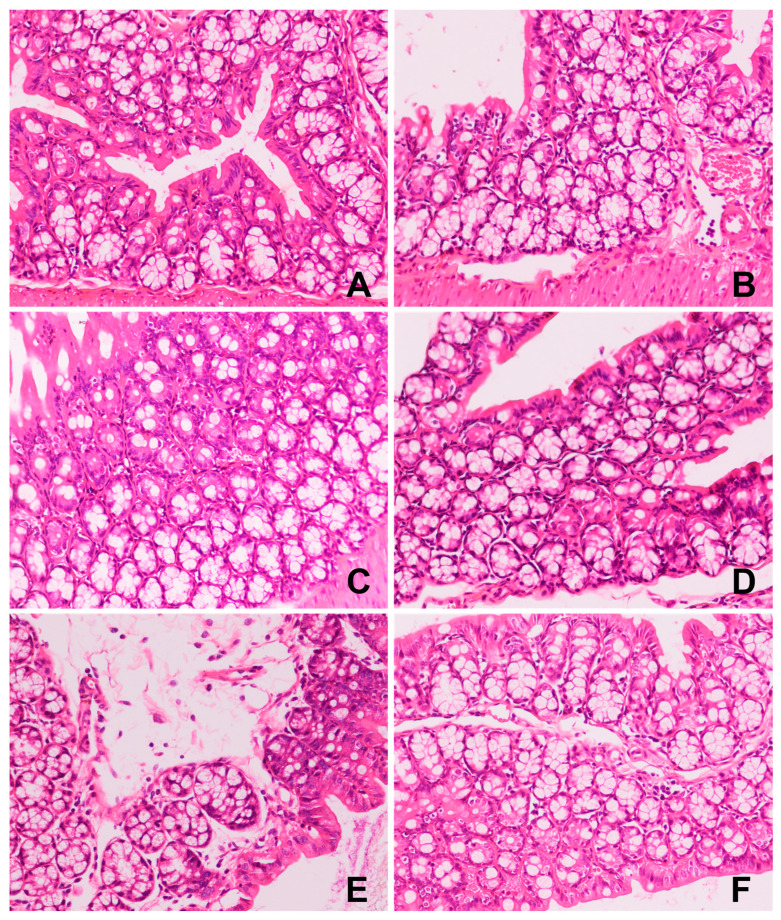
Effect of GFTTPs on histopathological observation of colon (200×). (**A**) NC; (**B**) MC; (**C**) 400C; (**D**) 200C; (**E**) 100C; (**F**) PC.

The histopathological observation (at 200× magnification) of colon in mice after administration with GFTTPs is shown in Figure 6. No evident abnormalities were observed in NC group, exhibiting a typical appearance: a well-preserved mucosa without ulcers, erosions, or any signs of inflammatory cell infiltration, primarily lymphocytes. The mucosal muscle also displayed no anomalies. Furthermore, neither the submucosa nor the muscle layer exhibited any congestion, hemorrhage, or edema.

However, in the MC group, mild infiltration by inflammatory cells, mainly lymphocytes and occasional neutrophils, were observed in the colonic mucosa. Additionally, there were signs of microvascular congestion in the colonic mucosa and lymph node hyperplasia in the submucosal layer. These results confirm the successful establishment of the constipation model. This observation aligns with other studies which highlighted reduced mucosal and muscle layer thickness and the infiltration of inflammatory cells into the damaged mucosa in loperamide-induced constipation models [30,39].

At the dose of 400 mg/kg, GFTTPs significantly improved colonic injuries in constipated mice, as evidenced by reduced inflammatory cell infiltration and lymph nodule formation (Figure 6F). Similar protective effects on the colon have been documented for polysaccharides from sources such as *Holothuria leucospilota* [39], *Spirulina platensis* [40], and *Chrysanthemum morifolium* [41].

For the 100C treatment group, the colon displayed partial inflammatory cell infiltration (mainly lymphocytes), coupled with microvascular congestion in the colonic mucosa (Figure 6D). In the 200C treated mice, mild inflammatory cell infiltration (mainly lymphocytes, occasionally neutrophils) was observed in the colonic mucosa, along with partial mucosal edema. Lymph node hyperplasia was observed in the submucosa. No lymph node hyperplasia was observed in the submucosa (Figure 6E). This result indicates that 400 mg/kg of GFTTPs exhibited a pronounced therapeutic effect, significantly ameliorating the colonic tissue alterations induced by loperamide hydrochloride.

#### 3.2.5. Effect of GFTTPs on Gut Microbiota

It is important to note that gut microbiota composition can vary significantly among individuals, influenced by a range of factors including genetics, diet, and environmental conditions. In our study, while GFTTP treatment consistently improved overall gut health and alleviated constipation symptoms, there was variability in the extent of gut microbiota modulation observed across different animals. This variability could be attributed to individual differences in baseline microbiota composition and responsiveness to dietary interventions. We observed that certain bacterial genera, such as *Lactobacillus* and *Coprococcus*, increased more markedly in some mice compared to others. These differences highlight the complex and individualized nature of gut microbiota responses, suggesting that while GFTTPs are generally effective, their impact may vary depending on the existing microbiota of the host. This observation underscores the importance of personalized approaches in dietary interventions aimed at modulating gut health.

The diversity, richness, and composition of gut microbial communities in mice administered with loperamide hydrochloride and/or GFTTP treatments were analyzed. The alpha-diversity metrics, namely Shannon, Simpson, Chao1, Good’s coverage, Observed species, Faith’s PD, and Pielou’s evenness, were measured based on OTUs with a sequence identity of ≥97%. These indexes are illustrated in Figure S2. Except Good’s coverage, there were no significant variations in the other parameters among the groups, indicating that loperamide induced constipation and negligibly impacted the diversity of microbial communities. Notably, the Chao1 matric showed marked differences in the MC group compared to the 400C, PC, and NC groups. This implies that the role of GFTTPs in alleviating constipation might stem from its regulation of the gut microbiota and overall intestinal micro-ecological environment.

**Figure 7:**
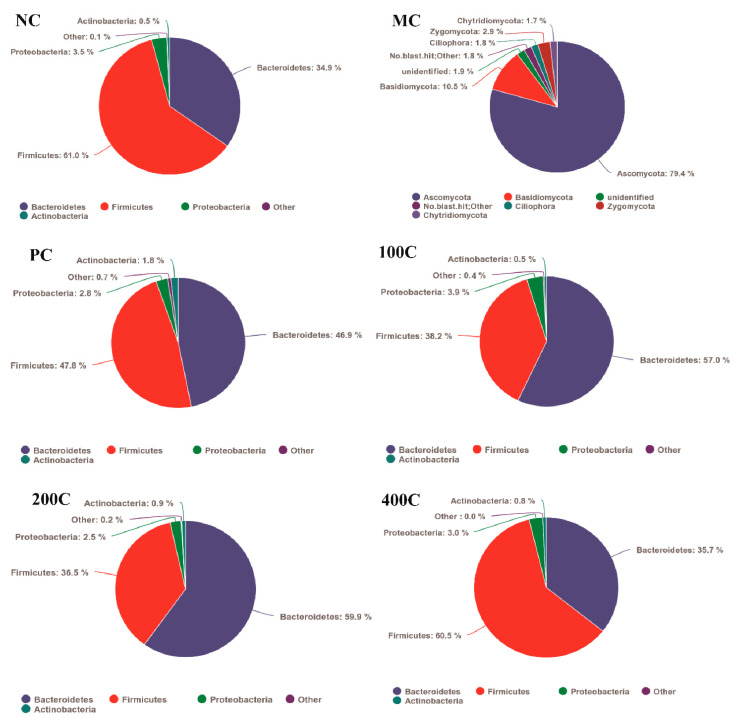
Relative abundance of major gut microbiota in mice at the phylum level.

As shown in Figure 7, at the phylum level, Firmicutes and Bacteroidetes belong to two predominant groups, collectively accounting for about 93–96% of the microbial community. Administering loperamide hydrochloride caused a notable shift in the relative abundance of these dominant bacteria. Urita et al. (2015) [42] reported a connection between an increase in Firmicutes and a decrease in Bacteroidetes with intestinal inflammation. Furthermore, they linked β-lactamase produced by clinically isolated Bacteroidetes strains to safeguarding other microbes in the gut.

Compared with the NC group, the relative abundance of Ascomycota and Basidiomycota in the MC group increased 79.4% and 10.5%, respectively. In the 100C group, we found a major rise in Bacteroidetes from 34.9% to 57%, and a decline in Firmicutes from 61% to 38.2% in the intestinal microbiota of mice. Similarly, in the 200C group treated with 200 mg/kg of GFTTP, the proportion of Bacteroidetes in the intestinal microbiota increased significantly from 34.9% to 35.7%, whereas Firmicutes decreased from 61% to 36.5%. Furthermore, the 400C group experienced a slight Bacteroidetes rise from 34.9% to 35.7%, and a tiny decline in Firmicutes from 61% to 60.5% when given 400 mg/kg of GFTTP. These findings hint at the potential of GFTTPs to augment the abundance of Bacteroidetes in the intestines of constipated mice. Supporting this, Zhang et al. (2022) [28] documented that constipated mice fed kiwi berry polysaccharide and polyphenol extracts exhibited a reduced Firmicutes presence but a heightened Bacteroidetes level in both colon and fecal microbiota.

**Figure 8:**
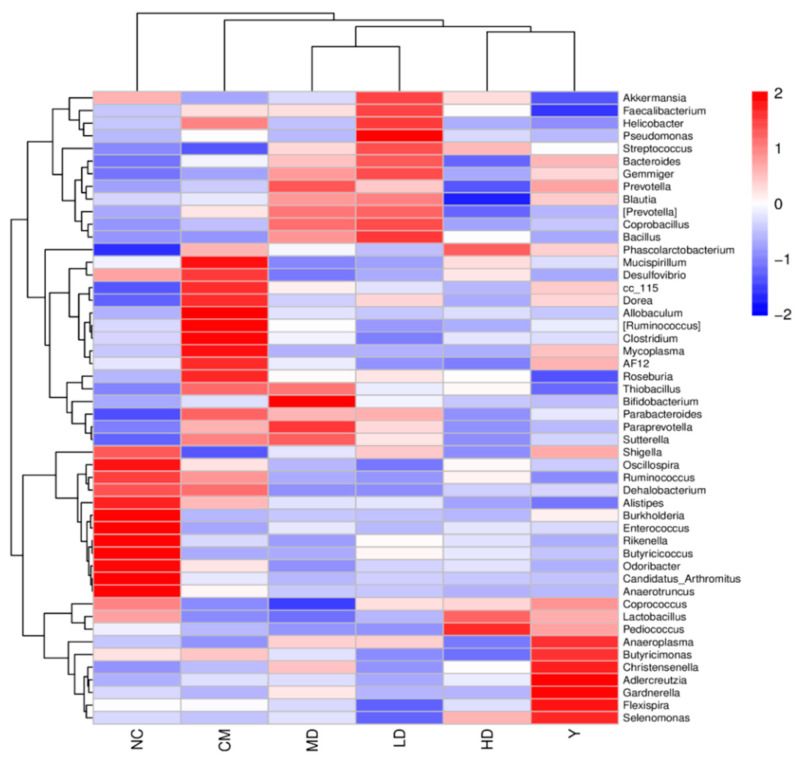
Heat map of the distribution of dominant groups at the genus level.

At the genus level, the relative abundance of bacteria was significantly different (Figure S3). The genus-level sequences *Lactobacillus*, *norank_f_Muribaculaceae*, *Bacteroides*, *Lachnospiraceae*, *Lachnospiraceae*_NK4A136_group and Helicobacter are the most abundant bacteria. In the MC group, several bacteria, including *Mucispirillum*, *Desulfovibrio*, cc_115, *Dorea*, *Allobaculum*, [*Ruminococcus*], *Clostridium*, *Mycoplasma*, AF12, *Roseburia*, *Parabacteroides*, *Sutterella* and *Dehalobacterium* were significantly increased compared to the NC group. However, after GFTTP administration, these microbial levels trended towards normalization (Figure 8).

Shi et al. (2023) [30] reported that the abundance of several bacteria, notably *Helicobacter*, *Alloprevotella*, *norank_f_Oscillospiraceae*, *Desulfovibrio Parabacteroides*, and *norank_f_Ruminococcaceae*, rose in constipated mice. Interestingly, *Desulfovibrio* was consistently observed in constipated mice. This bacterium has previously been associated with intestinal barrier damage and increased significantly in inflammatory bowel disease, resulting in apoptosis of gastrointestinal cells [43].

On the contrary, several bacterial genera, including *Shigella*, *Burkholderia*, *Enterococcus*, *Rikenella*, *Butyricicoccus*, *Odoribacter*, *Candidatus*_*Arthromitus*, *Anaerotruncus*, *Coprococcus*, *Lactobacillus*, and *Pediococcus*, were significantly decreased; levels of *Coprococcus*, *Lactobacillus*, and *Pediococcus* were recovered in the 400C group (Figure 8). *Coprococcus* is an important genus of the gut microbiota and is involved in carbohydrates’ fermentation [44]. Furthermore, *Lactobacillus* and *Pediococcus*, both Gram-positive and catalase-negative bacteria, primarily produce lactic acid as the main metabolic end product during carbohydrate fermentation, of which some strains were considered probiotics, underscoring their significance in gut health [45,46].

Regarding *Bacteroides*, both the NC and 400C groups exhibited lower levels compared to the MC group, indicating their reduced abundance. The PC, 200C, and 100C groups marked elevated levels of *Bacteroides* in comparison to the MC group. Additionally, the MC group showed augmented *Prevotella* levels, suggesting that constipation promotes intestinal inflammation in mice. Interestingly, both the NC and 400C groups indicated reduced *Prevotella* levels under the influence of 400 mg/kg GFTTP. This suggests the capacity of GFTTPs to restrain the proliferation of potentially pathogenic bacteria within the mouse gut. Thus, GFTTPs’ impact on the gut microbiota composition, particularly the increase in beneficial bacteria like *Lactobacillus* and *Coprococcus*, suggests a prebiotic-like effect that supports gut health through both direct and indirect biochemical interactions.

The NC group, which received no treatment, exhibited normal stool frequency, consistency, and gastrointestinal transit time, serving as the baseline for healthy mice. In contrast, the PC group, treated with phenolphthalein, showed significant improvements in these parameters compared to the model control (MC) group, demonstrating the efficacy of phenolphthalein as a standard laxative. When comparing the GFTTP treatment groups to the NC and PC groups, the 400 mg/kg GFTTP group showed improvements in stool frequency and consistency that were comparable to those observed in the PC group. However, unlike phenolphthalein, which primarily acts as a stimulant laxative, GFTTPs also modulated gut microbiota and upregulated aquaporins, providing a broader therapeutic effect. Furthermore, the GFTTP groups, particularly at 200 and 400 mg/kg, exhibited gastrointestinal transit rates similar to the NC group, indicating a return to normal physiological function. These comparisons highlight the potential of GFTTPs not only as an effective treatment for constipation, but also as a more holistic approach to gastrointestinal health.

#### 3.2.6. Effect of GFTTPs on SCFA Content

The concentrations of SCFAs including acetic acid, propionic acid, isobutyric acid, butyric acid, isovaleric acid, caproic acid, and valeric acid were evaluated in feces samples (Table 2). Results showed that the levels of these SCFAs in constipated mice were significantly diminished compared to the NC group (*p* < 0.05). With the exception of caproic acid, a noticeable increase in these SCFAs was observed after the administration of 400 mg/kg GFTTPs (*p* < 0.05).

Studies have shown that SCFAs play critical roles in various gut functions: they inhibit intestinal inflammation [47], enhance electrolyte and water metabolism [48], and promote the peptides related to gastrointestinal regulation [33]. These functions contribute to increased stool amount, reduced latency in passing the first black stool, improved fecal moisture, and overall constipation relief. They also accelerate intestinal motilities, prompt the expression of gene-encoding water channel proteins, and alleviate colon damage by histopathological examinations. All these observations suggest that GFTTPs have a pivotal role in mitigating constipation.

**Figure 9:**
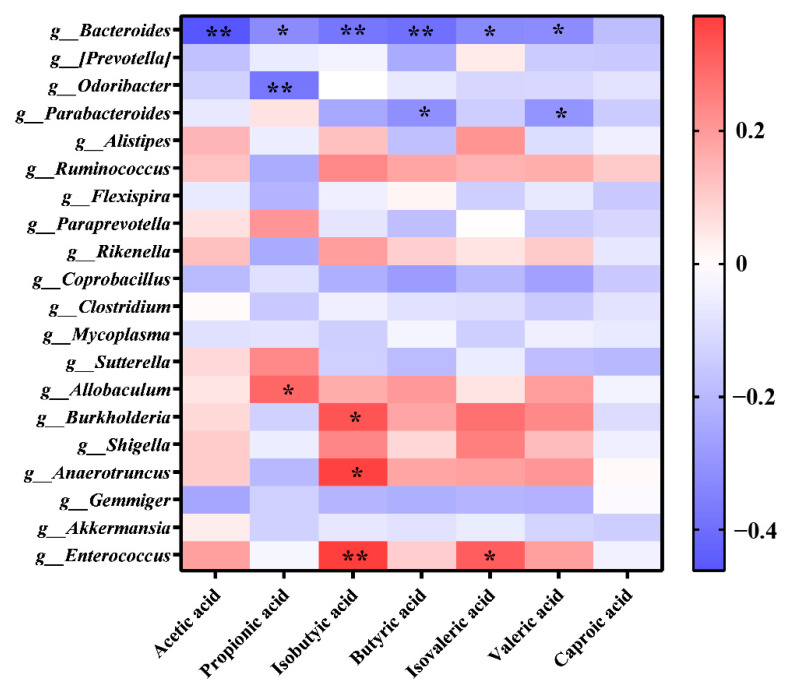
Differential analysis of the correlation between intestinal flora and SCFAs. ** indicated *p* ≤ 0.01, * indicated *p* ≤ 0.05.

It is well known that SCFAs are the main metabolites of dietary fiber fermented by carbohydrate intestinal microorganisms [22,30,40]. The correlation between colony composition and SCFA content in mice is presented in Figure 9. Caproic acid displayed a highly negative correlation (*p* < 0.01) with acetic acid, sobutyric acid, and butyric acid. Bacteroides showed a significant negative correlation (*p* < 0.05) with all six SCFAs, except for caproic acid. Bacteroides, capable of decomposing diet and mucosal polysaccharides, as well as those present on the surface of other gut microbes, thrive in the intestinal milieu [49]. Further, their ability to produce SCFAs helps to support the functionality and stability of the gut microbiota [50] by utilizing GFTTP. Additionally, isobutyric acid exhibited a significant positive correlation with bacteria like *Enterococcus*, *Anaerotruncus*, and *Burkholderia* (*p* < 0.05 or *p* < 0.01). This indicated that these bacteria potentially improved SCFA secretion, subsequently inhibiting the abundance of both *Odoribacter* and *Parabacteroides*. Indeed, we observed that *Odoribacter* exhibited a significant negative correlation with propionic acid (*p* < 0.01), and Parabacteroides demonstrated a significant negative correlation with butyric acid and avaleric acid (*p* < 0.05) (Figure 9). Thus, these data reasoned the possibility that GFTTPs, by enhancing the Bacteroides abundance and SCFAs secretion, play an important role in alleviating constipation in mice.

Above all, GFTTPs appear to modulate the expression of genes related to SCFA production, particularly those involved in the fermentation of dietary fibers by gut bacteria. SCFAs, such as butyrate, play a crucial role in maintaining gut health by serving as energy sources for colonocytes and regulating gut motility. Our data indicate that GFTTPs enhance SCFA production, which may contribute to its overall efficacy in relieving constipation.

## 4. Conclusions

Our study provides novel insights into the potential of Golden Flower Tibetan Tea Polysaccharides (GFTTPs) as a natural remedy for loperamide hydrochloride-induced constipation in mice. The findings demonstrate that GFTTP treatment can enhance water absorption and reduce fecal water content by upregulating the expression of aquaporins (AQP3 and AQP4) in the colon. This regulation of water transport is crucial in alleviating constipation symptoms and improving stool consistency and frequency.

Furthermore, GFTTPs play a pivotal role in modulating the composition of gut microbiota, promoting the proliferation of beneficial bacteria, such as Bacteroides, which enhances the stability and functionality of the gut ecosystem. This modulation is further supported by the increased production of short-chain fatty acids (SCFAs) through bacterial fermentation, which not only contributes to improved gut health but also supports the upregulation of genes associated with water channel proteins. GFTTPs’ ability to mitigate pathological tissue damage in the colon and accelerate intestinal motility further underscores its therapeutic potential.

These findings lend substantial support to the utilization of GFTTPs as a functional food ingredient for the dietary management of constipation. They underscore the significance of further exploration into the physicochemical properties, structural characteristics, and specific ameliorative mechanisms of GFTTPs concerning constipation. The dual mechanisms of action—regulating aquaporins and modulating gut microbiota—highlight the uniqueness of GFTTPs as a holistic approach to treating constipation. This research advances the field by offering a new perspective on the development of functional foods that provide both nutritional and therapeutic benefits.

However, while the results from this study demonstrate the potential efficacy of GFTTPs in alleviating constipation in mice, it is important to recognize that animal models do not fully replicate the complexity of human physiology. Differences in gut microbiota composition, metabolism, and overall gastrointestinal function between mice and humans may affect the applicability of these findings to human populations. Moreover, the long-term safety and efficacy of GFTTPs in humans remain to be fully evaluated. Future studies, including clinical trials, are necessary to determine whether the benefits observed in mice can be consistently reproduced in human subjects. These trials should also explore the potential for dose adjustments, as the optimal dose identified in mice may not directly translate to the most effective dose in humans. By conducting such studies, we can better understand the therapeutic potential of GFTTPs and their application in the dietary management of constipation in humans.

## Figures and Tables

**Table 1:** Basic physicochemical properties of GFTTP.

GFTTP	Content
**Chemical composition (%)**	
total sugar	17.83 ± 0.34
total phenols	9.24 ± 0.32
total protein	8.22 ± 0.48
**Molecular weight (Da)**	
Mw	70,989
Mn	24,227
Mp	66,395
**Monosaccharide composition (mol, %)**	
guluronic acid	2.138
mannuronic acid	0.054
mannose	6.786
ribose	0.486
rhamnose	4.389
glucuronic acid	1.086
galacturonic acid	17.177
glucose	1.875
galactose	32.212
arabinose	32.498
L-fucose	1.299

**Table 2:** Effects of GFTTPs on the concentrations of SCFAs in mice feces.

SCFAs	NC (μg/g)	MC (μg/g)	PC (μg/g)	100C (μg/g)	200C (μg/g)	400C (μg/g)
Acetic Acid	1537.655 ± 139.809 ^a^	916.917 ± 202.936 ^b^	1329.511 ± 149.902 ^a^	1239.255 ± 141.321 *	1348.105 ± 182.197 *^#^	1347.185 ± 183.901 *^#^
Propionic Acid	304.11 ± 66.245	198.07 ± 53.606 **	352.194 ± 70.846	312.844 ± 99.978	314.882 ± 178.344	403.203 ± 136.307
Isobutyric Acid	28.106 ± 11.662	15.331 ± 4.865 **	22.034 ± 7.971 **	23.915 ± 4.512 **	28.412 ± 10.181 **	16.356 ± 5.037 **
Butyric Acid	477.131 ± 187.69	237.539 ± 94.926	460.924 ± 205.229	352.185 ± 85.688	397.063 ± 117.726	411.467 ± 141.245
Isovaleric Acid	25.575 ± 9.303	18.428 ± 3.489 *	17.469 ± 6.622 *	19.968 ± 3.777 *	22.088 ± 6.716 *	14.821 ± 4.209 *
Caproic Acid	1.757 ± 0.592	2.374 ± 3.623	1.186 ± 0.197	1.21 ± 0.169	1.128 ± 0.171	1.199 ± 0.374
Valeric Acid	52.772 ± 16.175	9.136 ± 4.397 ***	18.679 ± 5.237 ***	17.443 ± 5.422 ***	24.395 ± 7.224 ***	17.9 ± 17.306 ***

Compare with the normal group: *** *p* < 0.001, ** *p* < 0.01, * *p* < 0.05; Compare with the model group: ^#^ *p* < 0.05. Different letters indicate a significant difference according to Duncan’s multiple range test (*p* < 0.05) between all groups.

## Data Availability

The original contributions presented in the study are included in the article, further inquiries can be directed to the corresponding author.

## Supplementary Materials

The following supporting information can be downloaded at: https://www.mdpi.com/article/10.3390/foods13172749/s1, Figure S1: Effect of GFTTP on the body weight gains of mice; Figure S2: Indexes of the alpha-diversity; Figure S3: Analysis of community composition based on genera level; Table S1: The primer sequences in the real-time quantitative PCR (RT-qPCR) analysis.
