# Supplementary material for: Golden Flower Tibetan Tea Polysaccharides Alleviate Constipation in Mice by Regulating Aquaporins-Mediated Water Transport System and Gut Microbiota
Source: Foods. 2024 Aug 29;13(17):2749. doi: 10.3390/foods13172749 (PMC11394950; doi:10.3390/foods13172749)
Supplement: Supplementary file 1 [file foods-13-02749-s001.zip › foods-3131361-supplementary.pdf]

Table S1 The primer sequences in the real-time quantitative PCR (RT-qPCR) analysis.

| Gene | Primer sequences 5´-3´ | Annealing temp. (°C) | Amplicon (bp) |
|---|---|---|---|
| M-AQP3-F3 | 5' AACCCCGCTGTGACCTTC 3' | 57.6 | |
| M-AQP3-R3 | 5' CCAAGTGTCCAGAGGGATAGG 3' | 58.5 | 220 |
| M-AQP4-F1 | 5' GATCAGCATCGCTAAGTCCG 3' | 58.3 | |
| M-AQP4-R1 | 5' TCCCAATCCTCCAACCACA 3' | 58.7 | 112 |
| M-AQP8-F1 | 5' TTGGGGAACATCAGCGGT 3' | 58.9 | |
| M-AQP8-R1 | 5' CTCCTGGACGATGGCAAAG 3' | 58.6 | 204 |

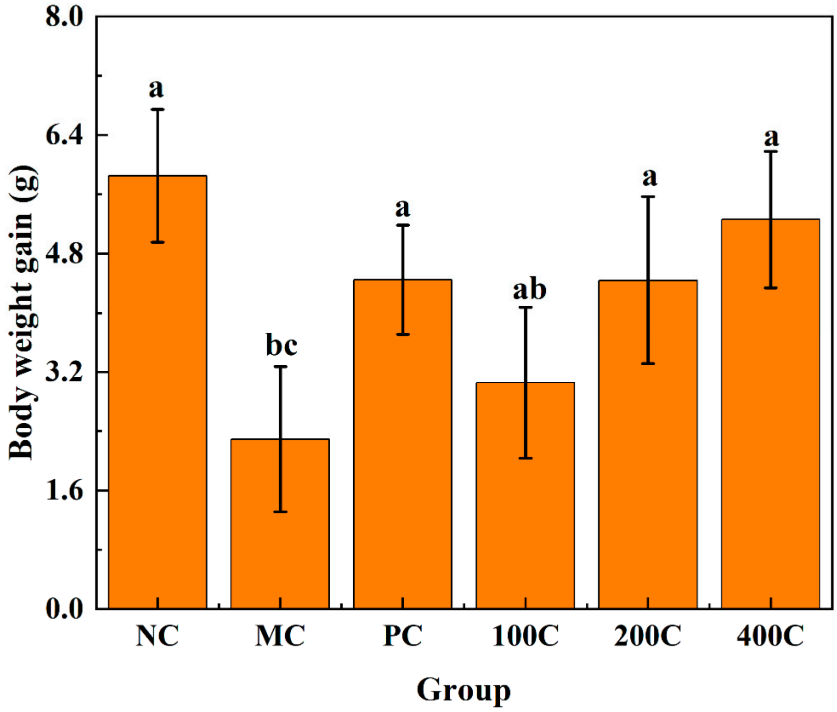


Figure S1 Effect of GFTTP on the body weight gains of mice.

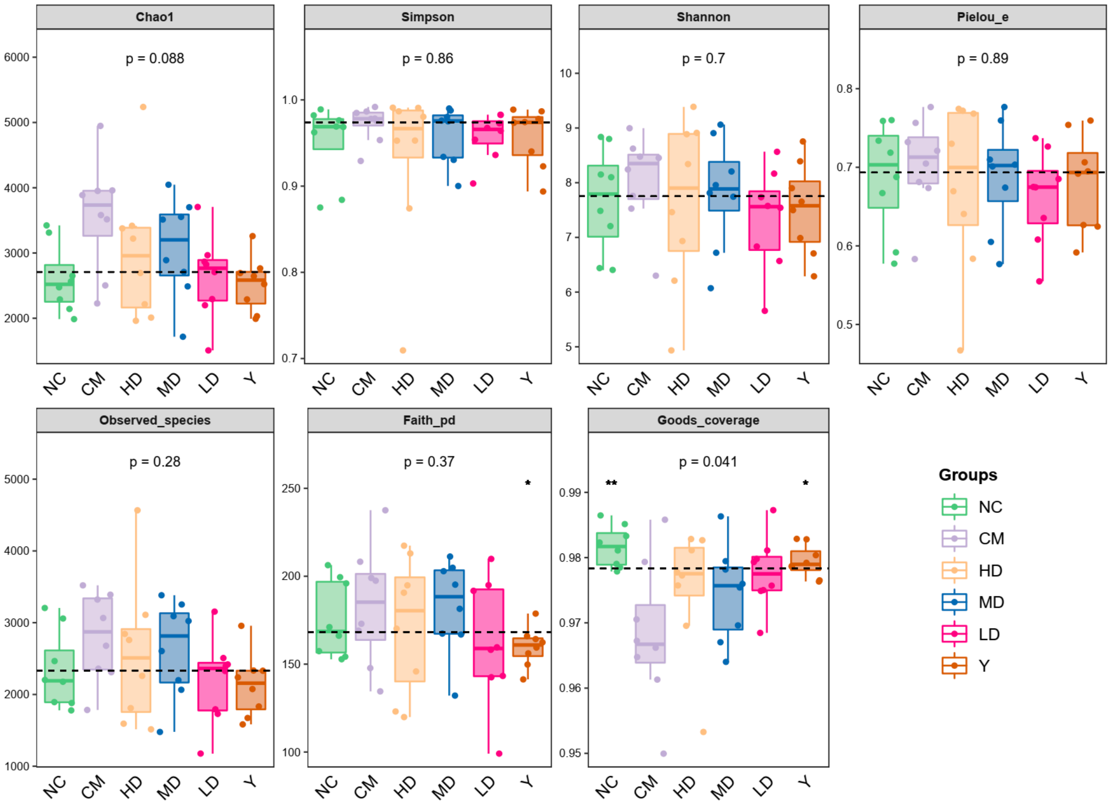


Figure S2 Indexes of the alpha-diversity.

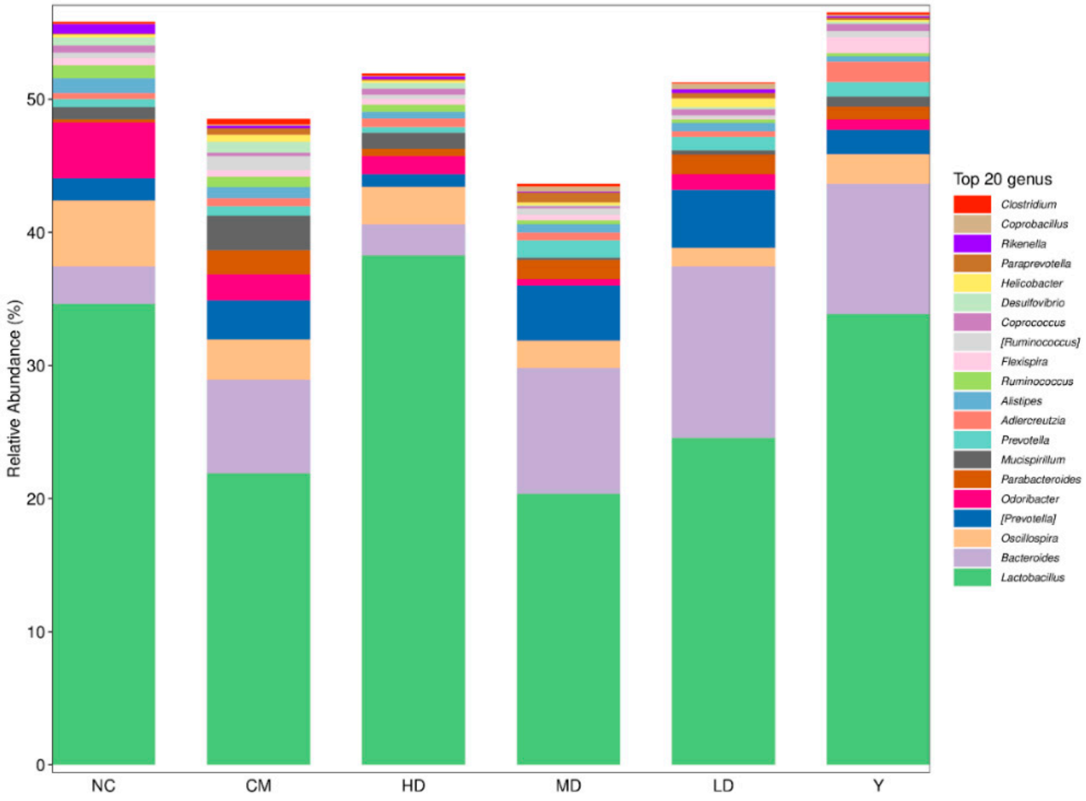


Figure S3 Analysis of community composition based on genera level.
